# Timely Hints for Dispensing Doctors

**Published:** 1913-09

**Authors:** 


Timely Hints for Dispensing Doctors. *Monthly Therapeutic Topics,* March, 1913. An Indiana physicians' supply house shipped some "Citrate of Caffeine" tablets labeled as containing 2 grains each. Analysis by the Bureau of Chemistry, U. S. Department of Agriculture, showed that these tablets contained less than ¾ of one grain of caffeine citrate.

The following are other statements from the same source regarding this firm's products: Compressed Calomel tablets labeled to contain 2 grains each, contained only 0.93 of a grain. Calomel and Soda tablets labeled to contain 1 grain each contained only 0.62 of a grain of calomel.

Compressed tablets Damiana Compound labeled to contain Phosphorus 1/30 gr., Extract Nux Vomica, ¼ gr., and Extract Damiana, 2 grains, contained only a trace of Phosphorus and less than 1/12 gr. Extract of Nux Vomica. Nitroglycerin tablets labeled to contain 1/50 grain contained less than 1/600 grain.

In the table below are the results of other findings by the Bureau of Chemistry on this and four other Indiana firms' products.

For detailed account of these frauds see notices of Judgment numbers 1796, 1799, 1810, 1843 and 1848, as issued by the Office of Secretary, U. S. Department of Agriculture.

| *Label Statement.* | *Actual Contents.* |
|---|---|
| Fluid extract Golden Seal, U. S. P. | Less than one-half |
| Wine Coca | Misbranded |
| Sodium Salicylate tablets, 3 gr. | 1.82 gr. |
| Strychnine Nitrate tablets, 1/40 gr. | 0.014 gr. |
| Nitroglycerin triturates, 1/100 gr. | 0.004 gr. |
| Acetanilid tablets, 5 gr. | 4.36 gr. |
| Nitroglycerin tablets, 1/50 gr. | 0.005 gr. |
| Acetphenetidin tablets, 3 gr. | 2.31 gr. |

Tablet triturates Aloin, 1/10 gr., Iron, 1 gr., Strychnine Sulph., 1/60 gr. . . . . . . . . . . . . . . . . . . . . . . . .0.01 gr. Strychnine
Tablets Ferruginous Blaud's, 3 gr., and Extract Nux Vomica, 1/6 gr. . . . . . . . . . . . . . . . . . . .1/16 gr. Ext. Nux Vomica
Tablets Flatulence, Ext. Nux Vomica, ¼ gr. Ext. Cascara Sagrada, 1 gr., Ginger, ¾ gr., Asafoetida, 1 gr., Diastase, 1 gr. . . . . . . . . . . . . . . . . . . . . .⅛ gr. Ext. Nux Vomica
Nitroglycerin tablets, 1/50 gr. . . . . . . . . . . . . . . . . . . . . . . .0.008 gr.
T. T. Ext. Nux Vomica, ¼ gr. . . . . . . . . . . . . . . . . . . . . . .1/75 gr.
Salol tablets, 2½ gr. . . . . . . . . . . . . . . . . . . . . . . . . . . . . . .1.8 gr.
T. T. Strychnine Nitrate, 1/40 gr. . . . . . . . . . . . . . . . . . . .1/50 gr.
Tablets Aloin, 1/5 gr., Ext. Belladonna, ⅛ gr., Ext. Nux Vomica, ⅛ gr. . . . . . . . . . . . . . . . . . . . . . . . . . . . . . . . . .
. . . . .¼ gr. Ext. Belladonna, 1/40 gr. Ext. Nux Vomica

